# Bitemarks analysis of orthodontically treated suspects – an identification approach

**DOI:** 10.1080/07853890.2021.1896921

**Published:** 2021-09-28

**Authors:** T. Nunes, S. Alves, J. Saraiva, J. Coelho, C. Caetano, A. Corte-Real

**Affiliations:** a Fifth-year student of Integrated Master in Dentistry, Faculty of Medicine, University of Coimbra, Coimbra, Portugal; b Faculty of Medicine, University of Coimbra, Coimbra, Portugal; c Forensic Dentistry Laboratory, Faculty of Medicine, University of Coimbra, Coimbra, Portugal

## Abstract

**Introduction:**

The advent of tridimensional (3D) technologies brings new and more reliable tools for bitemark analysis [1–7]. This experimental study intends to assess, in a forensic scenario, an identification approach for orthodontically treated suspects.

**Materials and methods:**

13 Cone Beam Computed Tomographic (CBCT) cranium files were selected from the clinical database of Coimbra Hospital and University Center/Faculty of Medicine of the University of Coimbra. The volunteer patients were recalled to bite an apple (golden delicious 75/80) which was immediately subjected to a CBCT scan. The 3D rendering of every bitemark was compared with the 3D upper dental arches obtained from the CBCT cranium scans of the simulated “suspects”. The research team was composed by 5 elements. The matching process consisted on corresponding landmark points in the bitemark and in the subjects’ dentition (upper dental arch). 169 comparisons were obtained between the 13 subjects and the 13 apples bitten. The kappa statistics analysis was performed.The study was approved by the Ethics Committee of Medicine’s Faculty of the University of Coimbra (CE-048/2017).

**Results:**

Cohen’s kappa values varied between 0.834 and 0.882. Fleiss kappa obtained a value of 0.604. The Friedman’s test was performed and the normality assumption was not verified (*p* > .05).

**Discussion and conclusions:**

The statistical analysis supports the accuracy and reliability of the methodology and the moderated agreement in classification, for the subject group. The post orthodontic treatment group can be included in bitemark sample in an identification scenario. It should not be an exclusion criteria for sample selection as it is usually performed. This experimental study opens up to new opportunities in forensic sciences regarding post orthodontic patients.
